# A good use of time? Providing evidence for how effort is invested in primary and secondary outcome data collection in trials

**DOI:** 10.1186/s13063-022-06973-8

**Published:** 2022-12-27

**Authors:** Heidi Gardner, Adel Elfeky, David Pickles, Annabel Dawson, Katie Gillies, Violet Warwick, Shaun Treweek

**Affiliations:** 1 Health Services Research Unit, University of Aberdeen, Health Services Research Unit, Foresterhill, Aberdeen, AB25 2ZD UK; 2 Division of Health Sciences, Warwick Medical School, University of Warwick, Coventry, UK; 3 Rheumatology Department, Leeds Teaching Hospitals NHS Trust, Leeds, UK; 4 Durham, UK; 5 School of Medicine, University of Dundee, Dundee, UK

**Keywords:** Randomised trials, Data collection, Outcomes, Core outcome sets, Research waste

## Abstract

**Background:**

Data collection is a substantial part of trial workload for participants and staff alike. How these hours of work are spent is important because stakeholders are more interested in some outcomes than others. The ORINOCO study compared the time spent collecting primary outcome data to the time spent collecting secondary outcome data in a cohort of trials.

**Methods:**

We searched PubMed for phase III trials indexed between 2015 and 2019. From these, we randomly selected 120 trials evaluating a therapeutic intervention plus an additional random selection of 20 trials evaluating a public health intervention. We also added eligible trials from a cohort of 189 trials in rheumatology that had used the same core outcome set.

We then obtained the time taken to collect primary and secondary outcomes in each trial. We used a hierarchy of methods that included data in trial reports, contacting the trial team and approaching individuals with experience of using the identified outcome measures. We calculated the primary to secondary data collection time ratio and notional data collection cost for each included trial.

**Results:**

We included 161 trials (120 phase III; 21 core outcome set; 20 public health), which together collected 230 primary and 688 secondary outcomes. Full primary and secondary timing data were obtained for 134 trials (100 phase III; 17 core outcome set; 17 public health). The median time spent on primaries was 56.1 h (range: 0.0–10,746.7, IQR: 226.89) and the median time spent on secondaries was 190.7 hours (range: 0.0–1,356,832.9, IQR: 617.6). The median primary to secondary data collection time ratio was 1.0:3.0 (i.e. for every minute spent on primary outcomes, 3.0 were spent on secondaries). The ratio varied by trial type: phase III trials were 1.0:3.1, core outcome set 1.0:3.4 and public health trials 1.0:2.2. The median notional overall data collection cost was £8015.73 (range: £52.90–£31,899,140.70, IQR: £20,096.64).

**Conclusions:**

Depending on trial type, between two and three times as much time is spent collecting secondary outcome data than collecting primary outcome data. Trial teams should explicitly consider how long it will take to collect the data for an outcome and decide whether that time is worth it given importance of the outcome to the trial.

**Supplementary Information:**

The online version contains supplementary material available at 10.1186/s13063-022-06973-8.

## Introduction

Trials are one of the most rigorous ways of testing treatments, but they can be both expensive and time consuming [1]. Increasingly complicated trial protocols contribute to this [2, 3]. While some of this complexity may be inherent to the way an intervention has to be evaluated, there is often scope for trialists to reduce complexity (and therefore work) without threatening the usefulness of the trial.

Outcome selection, and the data that must be collected to report outcomes, is an area where trialists almost always have scope to make changes to reduce complexity and work. In addition to the work done by participants and clinical staff to provide data, the trial team itself must do work to create data collection forms, build a data management system to store them, oversee and update that system, ensure that data are stored securely and then check and clean data in preparation for analysis. It is not surprising that it is estimated that over 30% of all staff work hours spent on trials is by data managers [4].

How these hours of work are spent is important because outcomes are not created equal: participants, trial teams, the public, funders and other trial stakeholders are interested in some more than others. Trial teams themselves declare one outcome (or occasionally a few) to be the most important outcome and call it the primary outcome. The primary outcome generally drives the size of the trial and future judgements as to whether the trial intervention is effective are largely framed around the primary outcome. All other outcomes are then, by definition, of less importance and are widely known as secondary outcomes.

We might expect the distribution of trial data collection effort to be more or less in line with the importance of the data being collected, but this is generally not the case. For example, a 2015 study undertaken in the USA looking at 15 pharmaceutical companies and 116 protocols found that for phase III (i.e. later stage, definitive) trials, 7% of data collection items were linked to primary outcomes, 36% to key secondaries, 32% with basic medical history and 25% had nothing to do with the trial research questions and supported supplemental secondary, tertiary and exploratory end points [3]. Of course, some of these data may be important for hypothesis generating purposes as this information would be difficult, in some cases impossible, to collect in another setting. That said, it could be argued that 25% of all data collection items is excessive when the cost of collecting these data was an estimated $3.7 billion annually in the USA in 2015 [3]. Crowley and colleagues counted the items on data collection forms in 18 trials and found that primary outcome data accounted for a median of 5% of all items collected, compared to a median of 40% for secondary outcomes [5]. Non-outcome data such as participant identifiers and demographic data accounted for a median of 33% of all data items.

Despite this data collection work, substantial amounts of collected data remain hidden. A Cochrane systematic review comparing entries in trial registries to published trial reports found that 10% to 18% of primary outcome data and 44% of secondary outcome data were not published [6]. In a review of all trials submitted to a German ethics committee between 2000 and 2002, Kirkham et al. found that only 47% of the two and half million items of outcome data collected from participants in these 308 trials were fully published [7]. Heneghan et al. give a catalogue of problems with data collection, including lack of relevance to decision-makers and poorly specified and collected data [8].

Irrelevant data collection is a problem for all trials but particularly phase III trials, intended as they are to be of direct relevance to decision-makers. By the start of a phase III trial, there ought to be little doubt as to what future users of the results need nor much energy spent collecting information they are not interested in. Increasing the data collection workload will increase the burden of participation, which for phase III trials may affect hundreds if not thousands of people (both those taking part and those delivering the trial). While exploratory data collection might be reasonable, essential even, in early-phase trials, collection of exploratory data in phase III trials needs careful attention. The trial team has defined a clear focus of attention—the primary outcome(s)—and it is on the primary outcomes that the trial will be judged. Substantial exploratory data collection in phase III trials runs the risk of overwhelming participants and trial team alike and threatening the purpose of the trial: to give decision-makers the information they need to make better decisions.

The ORINOCO study aimed to increase awareness among trialists of how data collection effort (measured in time and the cost of that time) is distributed across outcomes collected in trials intended to be of direct relevance to decision-makers. The project had three phases; in this paper, we describe work completed in just the first two phases to answer a single question:

Across a random selection of trials, how much time is spent collecting primary outcome data compared to time spent collecting secondary outcome data?

This work is part of the Trial Forge initiative to improve trial efficiency (https://www.trialforge.org).

## Methods

The protocol for ORINOCO is available at https://osf.io/FNB3E/.

### Stage 1: Identifying trials and outcomes

We used three different search strategies to create our cohort of trials; these strategies are given in Supplementary File 1.

Our first search targeted trials indexed by PubMed between 2015 and 2019. From this list, we then used Microsoft Excel’s random number generator to randomly select trials to which we then applied the following criteria:

- The trial must be a ‘late stage’ trial (often called a phase III trial)
- Trials in any disease area
- Conducted in any country
- Non-commercial. By non-commercial, we mean trials that are not funded and run by commercial organisations such as pharmaceutical companies. A trial that is commercially funded but run by an academic team was eligible
- Trial interventions must aim to impact a health-related outcome
- Should not be a feasibility study

We called this sample ‘phase III’ trials, and we continued to randomly select trials until we had 120 that met our criteria. The figure of 120 trials is purely pragmatic; we aimed to include ‘enough’ trials to provide compelling data given the time and resources we had available to us. To ensure a spread of trials across different disease areas, we added a rule: a maximum of 15% of the phase III trials selected (i.e. 18 trials) can be from one broad disease category (e.g. oncology, diabetes). If this threshold was reached, no further trials in that disease area would be included.

Our second search targeted trials that used a core outcome set (‘core outcome set’). A core outcome set is an agreed standardised selection of outcomes which should be measured and reported, as a minimum, in all trials for a specific clinical area [9]. Core outcome sets are recommended by the UK’s National Institute for Health and Care Research [10], but the impact on workload of mandating a set of outcomes for a trial is unclear. Our aim was to select 20 core outcome set trials by randomly selecting trials that met our eligibility criteria (i.e. the bullet list above) from the 189 completed trials that used the rheumatoid arthritis core outcome set in Kirkham et al. [11]. This core outcome set is one of the most mature, coming as it does from OMERACT, an international network initiated in 1992 to improve outcome measurement in rheumatology [12]. Kirkham’s review was an efficient way of identifying trials that had used the set. If any of the other 120 phase III trials used core outcomes, we planned to add them to our group of core outcome trials.

Finally, our third search targeted public health trials (‘public health’), of which we wanted 20. We did this because we thought they may be substantively different to other types of trials when it comes to data collection, particularly around collection of multiple behavioural measures over extended periods. We used the Excel-based method of random selection, and to ensure a spread of trials across different public health issues, we added a rule that a maximum of 25% (i.e. four trials) of the trials selected can be from one broad disease category.

Again, the decision to include 20 trials using a core outcome set, and 20 trials evaluating a public health intervention is pragmatic. We anticipated that these types of trials may provide a different perspective on data collection, with the potential for different ways of distributing data collection effort compared to our general phase III trials group.

Together, this gave a target of 160 trials, a balance between enough trials to provide compelling data and a sample that is unmanageably large given the resources available. For each trial, we identified the primary and secondary outcomes and the measurement instruments used to collect them. We obtained the protocol or trial registration entry for as many as we could to overcome the potential problem of incomplete reporting in trial reports. Where there were unexplained differences between documents, we used the longer list of outcomes.

Once we had a list of trials that met our inclusion criteria (stage 1), we simultaneously identified outcomes (stage 1) and obtained timings for each of those outcomes (stage 2, more information below). Stage 1 and Stage 2 of our methods overlapped, rather than being completely distinct. This was a pragmatic decision to allow us to ensure we could begin the process of obtaining timings for each outcome as soon as possible. We knew this process would require significant time investment and spending time identifying the outcomes in stage 1 before moving on to obtaining timings would have impacted our project timeline considerably.

### Stage 2: Obtain timings for each outcome

For each of the primary and secondary outcome measures coming from stage 1, we needed an assessment of how long it took to collect the data required by the measure. We called this ‘*Time to collect*’ for the outcome.

To calculate *Time to collect*, we used a hierarchy of methods based on those used in our pilot work [13]

1. *Investigate the published trial paper, protocol, or registration*—if timing information was provided in one of the trial publications, we would use this.
2. *Ask the trial team*—using a standard template we emailed the corresponding author listed on the published paper, as well as the chief investigator and the trial manager where these details were available. A reminder email was sent two weeks after the initial contact if no member of the team responded.
3. *Web-based resources*—such as the Shirley Ryan Ability Lab Rehabilitation Measures Database (https://www.sralab.org/rehabilitation-measures) and Improving Long-Term Outcomes Research for Acute Respiratory Failure (https://www.improvelto.com/).
4. *Contacting professional networks*—this was done through our network of trial colleagues (e.g. trial managers) who we thought may have used an outcome, or at least have an idea of how long using the outcome might take. We also opportunistically approached people at the Evidence Live 2019, Oxford, UK conference regarding outcomes for we lacked timing data.

Where different trials used the same outcome measure (e.g. two trials used SF-36 to measure quality of life), we aimed to get timings from each trial team. We also recorded the number and timing of measurement points. We did not set a minimum *Time to collect*; we took whatever trial teams gave us.

Finally, it is worth noting that our *Time to collect* timings represent *only* data collection time, not total data collection effort. They do not include time spent designing and creating the data management system, managing the data collected, doing data quality assurance, data analysis, or time spent by participants travelling to and from measurement sites to provide data. To take a concrete example, if a trial team took a blood sample, *Time to collect* is the time taken by a member of staff to draw the blood sample from a participant and nothing else.

### Analysis

We calculated the total time spent collecting outcome data for each trial by multiplying *Time to collect* for each outcome by the number of trial participants and the number of times the outcome was measured. This gave total *Time to collect* for that trial. We assumed 100% retention at each measurement point because we were interested in the maximum workload that a trial team had committed to with their design. We also calculated the ratio of time spent collecting primary and secondary outcome data.

To give an indication of how much a trial’s data collection work cost, we calculated a notional cost based on a single member of staff being needed for all measurements and used the February 2022 hourly rate for a UK National Health Service research nurse of £23.51 [14]. The data collection cost per trial was then calculated by multiplying the total *Time to collect* in hours by the hourly rate. We acknowledge that these costs are unlikely to be exact for any trial in our sample, that some trials will need more or fewer members of staff for measurements and that some costs will be borne by participants rather than staff. The costs are intended to give ballpark, comparative figures to promote discussion.

## Results

### Identifying trials

We included a total of 161 trials, which included two deviations from our protocol for the core outcome set trials. Firstly, we chose to include all 21 eligible trials in Kirkham et al. [11] rather than randomly select 20 of them. Secondly, the trials in Kirkham et al. included some phase IV trials, and we decided to include these because they were all definitive evaluations with the aim of immediate clinical relevance, which is what we were interested in. Not all the trials in Kirkham et al. had a published report, with Kirkham et al. working with trial registration entries where this was the case. We searched for full texts in case there were reports published after Kirkham’s review and we found one such trial report, which we then used in ORINOCO. There were no trials in our phase III and public health groups of trials using core outcome sets, which means that the entire core outcome set group of trials were identified from Kirkham et al.

Of the 161 trials, 120 were phase III, 21 were core outcome set, and 20 were public health trials (Table 1 (minutes) and Table 2 (hours); the trials are listed individually in Supplementary File 2). Between them, the 161 trials had a total of 230 primary outcomes and 688 secondary outcomes.

**Table 1:** Number of included phase III, core outcome and public health trials, together with the total number of data points included in our calculations for both primary and secondary outcomes, the interquartile ranges including upper and lower limits, median total trial data collection time (i.e. total *Time to collect*; in minutes) for each trial type and the median primary to secondary *Time to collect* ratio for each trial type. Note: two trials did not contribute to the ratio calculations (one phase III, one core outcome set) because the primary outcome *Time to collect* was 0 for both (i.e. including these led to a division by zero error)

Included trials with full ***Time to collect*** data								
	Number of trials	Number of primary outcomes data points	Median total trial time to collect for primary outcomes (minutes) [min–max]	Primary outcome IQR (minutes) [lower limit–upper limit]	Number of secondary outcomes data points	Median total trial time to collect for secondary outcomes (minutes) [min–max]	Secondary outcome IQR (minutes) [lower limit–upper limit]	Median primary to secondary total ***Time to collect*** ratio
All trials	134	134	3367.5 [0.0–644,800.0]	13,613.3 [− 19,089.38–35,363.63]	134	11,440.0 [0.0–81,409,972.0]	37,057.5 [− 52,586.25–95,643.8]	1: 3.0
Phase III	100	100	3225.0 [0.0–644,800.0]	7684.0 [− 10,218.5–20,517.5]	134	9984.00 [0.0–81,409,972.0]	28,400.5 [− 39,650.8–73,951.3]	1: 3.1
Core outcome set	17	17	2500.0 [0.0–120,285.0]	10,395.0 [− 14,890.5–26,689.5]	17	9475.0 [90.0–75,600.0]	19,013.0 [− 26,236.5–49,815.5]	1: 3.4
Public health	17	17	21,231.5 [410.0–363,168.0]	44,916.0 [− 64,365.0–115,299.0]	17	30,250.0 [546.0–451,710.0]	98,208.0 [− 139,324.0–253,508.0]	1: 2.2

Number of included trials (overall): phase III—120; core outcome set—21; public health—20

Total: 161

**Table 2:** Number of included phase III, core outcome and public health trials, together with the total number of data points included in our calculations for both primary and secondary outcomes, the interquartile ranges including upper and lower limits, median total trial data collection time (i.e. total *Time to collect*; in hours) for each trial type and the median primary to secondary *Time to collect* ratio for each trial type. Note: two trials did not contribute to the ratio calculations (one phase III, one core outcome set) because the primary outcome *Time to collect* was 0 for both (i.e. including these led to a division by zero error)

Included trials with full ***Time to collect*** data								
	Number of trials	Number of primary outcomes data points	Median total trial time to collect for primary outcomes (minutes) [min–max]	Primary outcome IQR (minutes) [lower limit–upper limit]	Number of secondary outcomes data points	Median total trial time to collect for secondary outcomes (minutes) [min–max]	Secondary outcome IQR (minutes) [lower limit–upper limit]	Median primary to secondary total ***Time to collect*** ratio
All trials	134	134	56.13 [0.0–10,746.67]	226.89 [− 318.16–589.39]	134	190.67 [0.0–1,356,832.87]	617.63 [− 876.44–1594.06]	1: 3.0
Phase III	100	100	53.75 [0.0–10,746.67]	128.07 [− 170.31–341.96]	134	166.40 [0.0 –1,356,832.87]	473.34 [− 660.85–1232.52]	1: 3.1
Core outcome set	17	17	41.67 [0.0–2004.75]	173.25 [− 248.18–444.83]	17	157.92 [1.50–1260.00]	316.88 [− 437.28–830.26]	1: 3.4
Public health	17	17	353.86 [6.83–6052.80]	748.60 [− 1072.75–1921.65]	17	504.17 [9.10–7528.50]	1636.80 [− 2322.07– 4225.13]	1: 2.2

### *Time to collect* for each outcome

Getting *Time to collect* data proved challenging. Out of 161 trials, 88 trial teams supplied all or some timing data for primary outcomes. Eighty-six trial teams supplied all or some timing data for secondary outcomes, and of those, 74 trial teams supplied all or some timing data for both primary and secondary outcomes. We managed to get full primary and secondary timing data for 134 of the 161 trials, of which 100 were phase III, 17 core outcome and 17 public health trials (see Supplementary File 2). Poor reporting in trial manuscripts meant that we were sometimes unable to identify one or all of primary outcomes, secondary outcomes, the measurement instruments used to collect outcomes, the number of participants or the number of times outcomes were collected. In these cases, trial registry entries sometimes gave more information but if not, we emailed members of the trial team to confirm that our assumptions about their outcomes were correct.

Some trial teams gave us time details as a range (e.g. ‘A blood test usually takes between 5.0 and 10.0 min’), and we used the mid-point of the two timings provided by the team (in this case, 7.5 min). Alternatively, some teams gave timings for a range of outcomes, e.g. a 45-min appointment would cover two primary outcomes and four secondary outcomes. Where possible, we then substituted data from elsewhere in the database to improve the accuracy of the timing estimate and then split the rest of the time by the number of outcomes. In other cases, trial teams were unable to estimate how long collecting all or some individual outcomes took, but they were able to estimate total data collection time per measurement visit. When this happened, we divided the total time equally between the outcomes. Where possible, we used timing data collected from other trial teams to improve the accuracy of these figures.

#### Primary and secondary outcome collection times

Table 1 (minutes) and Table 2 (hours) show the median time spent collecting primary and secondary outcome data for the 134 trials with full *Time to collect* data (i.e. we have primary and secondary *Time to collect* plus the number of times outcomes were measured and the number of participants randomised). Across all trials, the median time spent collecting primary outcome data is 56.1 h (range 0.0 to 10,747.67, IQR: 226.89), and for secondary outcomes, it is 190.7 h (range 0.0 to 1,356,834.0, IQR: 617.6).

Most data collection was bespoke to the trial but there was use of routine medical records. These could make a dramatic difference to data collection time for some outcomes. For example, Trial ID 122, a phase III prostate cancer screening trial, used the UK’s NHS Digital system (https://digital.nhs.uk/data) to provide data for its primary outcome, giving a *Time to collect* of 0.0 for that outcome. The trial had four secondary outcomes, one of which came from NHS Digital (0.0 min) and two others from medical record reviews, which together took a total of six minutes. The fourth secondary outcome was health-related quality of life, which was assessed using a battery of measures taking 43.0 min. Our discussions with members of trial teams that used medical records for data collection led us to settle on 3 min per item for chart review. Chart review time could add up: e.g. Trial ID 151, a phase III reproductive health trial, had 25 secondary outcomes needing chart review or 75.0 min per participant.

The total primary outcome data collection times for each trial are given in Supplementary File 3, with similar data for secondary outcomes in Supplementary File 4.

#### Ratio of primary to secondary outcome collection times

Table 1 (minutes) and Table 2 (hours) also show the median ratio of time spent collecting primary and secondary outcome data. Note that two trials (Trial ID 5 and Trial ID 122) had primary outcome *Time to collect* of 0.0, meaning they are not included in the ratio calculations. The overall median primary to secondary ratio is 1.0:3.0. In other words, for every hour spent collecting primary outcome data, 3 h were spent on secondary outcomes. The median primary to secondary ratio was similar for phase III trials (1.0:3.1) and core outcome set trials (1.0:3.4) but lower for public health trials (1.0:2.2).

**Fig. 1:**
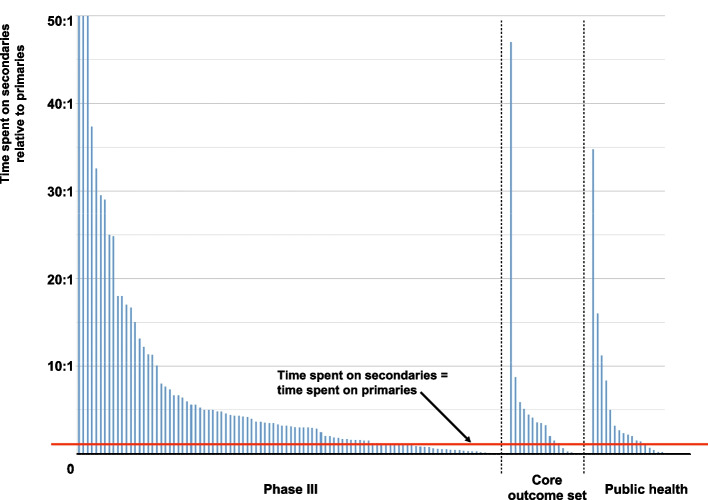
Time spent on secondary outcomes relative to primaries for phase III, core outcome and public health trials. Note that the vertical axis is truncated at a ratio of 50:1

Figure 1 shows the time spent collecting secondary outcome data relative to that spent on primaries for the three trial types. The vertical axis is truncated: three phase III trials spent more than 50 times as much time collecting secondary outcome data than primary (Trial ID 48, a phase III drug trial, spent 401 times as much; Trial ID 120, a phase III surgical trial, spent 62 times as much; and Trial ID 99, a phase III behaviour change trial, spent 60 times as much).

#### Estimated costs of primary to secondary outcome collection

Table 3 gives median notional data collection costs. The median overall data collection cost was £8015.73 based on the assumption that a single member of staff was needed for all measurements. There was a substantial range of £52.90 (Trial ID 38, a core outcome drug trial) to £31,899,140.70 (Trial ID 122, a very large phase III prostate cancer screening trial). Median overall data collection costs are similar for phase III trials (£7096.30, IQR: £16,581.51) and core outcome set trials (£8901.67, IQR: £21,119.82) but are much higher for public health trials (£20,043.45, IQR: £36,841.15). The full costing data for the 134 individual trials is given in Supplementary File 5.

**Table 3:** Median notional costs in GBP Sterling (£) of primary, secondary and total data collection costs for phase III, core outcome set and public health trials, including number of included phase III, core outcome and public health trials, together with the total number of data points included in our calculations for both primary, secondary, and all outcomes, the interquartile ranges including upper and lower limits. Note: two trials did not contribute to the ratio calculations (one phase III, one core outcome set) because the primary outcome *Time to collect* was 0 for both (i.e. including these led to a division by zero error)

	Number of trials	Number of data points included	Median notional cost for total primary outcome collection [min–max]	IQR notional cost for total primary outcome collection [lower limit–upper limit]	Median notional cost for total secondary outcome collection [min–max]	IQR notional cost for total secondary outcome collection [lower limit–upper	Median notional total cost for total data collection [min–max]	IQR notional cost for total data collection [lower limit–upper
All included trials with full *Time to collect* data	134	134	£1319.50 [£0–£252,654.13]	£5307.09 [£4525.09–£14,049.72]	£4482.57 [£0–£31,899,140.70]	£14,329.35 [£12,566.10–£37,586.61]	£8015.73 [£52.90–£31,899,140.70]	£20,096.64 [£17,091.62–£53,246.62]
Phase III	100	100	£1263.66 [£0–£252,654.13]	£3010.85 [£2242.36–£8295.60]	£3912.06 [£0–£31,899,140.70]	£11,430.02 [£9696.16–£30,308.92]	£7096.30 [£122.25–£31,899,140.70]	£16,581.51[£13,832.45–£44.202.82]
Core outcome set	17	17	£979.58 [£0–£47,131.67]	£4073.11 [£3660.51–£10,595.37]	£3712.62 [£35.27–£29,622.60]	£7449.93 [£6108.09–£19,966.65]	£8901.67 [£52.90–£48,295.42]	£21,119.82 [£19,362.44–£54,556.91]
Public health	17	17	£8319.21 [£160.65–£142,301.33]	£17,599.59 [£15,831.05–£45,767.50]	£11,852.96 [£213.94–£176,995.04]	£38,481.17 [£33,786.22–£100,897.87]	£20,043.45 [£427.88–£252,850.05]	£36,841.15 [£17,207.36–£111,736.67]

## Discussion

Our study shows that, generally speaking, trial teams spend about three times more of their data collection time collecting secondary outcome data than they do collecting primary outcome data. Or put another way, for every hour spent collecting primary outcome data, 3 h are spent collecting secondary outcome data. This is not confined to any one disease area; our trial portfolio includes trials across over 40 clinical disciplines with representation from over 40 countries. A full list of trials including details of clinical discipline, country, year and number of participants randomised is available in Supplementary File 2.

We did not find any clear suggestion that using a core outcome set increases this ratio. Public health trials balance their data collection time between primary and secondary outcomes more evenly although total data collection time, and therefore cost, is noticeably greater than for other trials.

It is important to note that all our timing and therefore cost data consider only the act of data collection itself (e.g. doing the measurement, or filling in the form) and not everything that follows on from that (e.g. creating and maintaining a data management system to handle the data, getting the recorded data into that data management system and chasing queries when data are inconsistent or missing). It is also worth emphasising that our costs are notional: we have assumed one member of staff is needed and costed on that basis. We do not know how many staff were needed or how much data collection actually cost for any trial. As trial teams struggled to provide their timing data, we are confident that in the vast majority of cases trial teams themselves do not know how much data collection in their trial actually cost either. Our costs are indicative only and should be judged with that in mind.

Allocating most data collection effort to secondary outcomes is not a problem if (a) this work is justified given the known information needs of the intended users of the results and (b) this activity is planned and budgeted for. Literature and experience suggest that these criteria are not always met. Heneghan and colleagues [8] cite numerous examples of the lack of relevance of trial outcome data to patients and other decision-makers, including the use of surrogate and composite outcomes. The outcomes trialists choose are not always the ones patients consider most important [15]. Recent work by Trial Forge comparing the outcome 44 trial teams chose as their primary outcome with what patients and healthcare professionals ranked as the most important outcome found that they agreed just 30% of the time [16]. That many trial teams struggled to tell us how long it took them to collect their data strongly suggests that resource planning and budgeting does not explicitly account for data collection workload. Allocating most data collection effort to secondary outcomes is rarely a considered judgement, it just happens.

We might accept some of this if all trials finished on time, to budget and published all their data but we know this is not true. Recruitment and retention problems are widespread [17, 18], the cost of trials is escalating [1] and substantial amounts of data never make it into the public domain [6]. A study evaluating trials funded by the UK’s National Institute for Health Research Health Technology Assessment Programme, a highly competitive funding stream, found that between 1997 and 2020, 128/388 (33%) of trial teams needed to extend their recruitment period [17]. Only 207/388 (53%) reached 100% of the original recruitment target and median retention for the primary outcome was 88%. There were no data on secondary outcome retention, but it was probably lower. Even experienced teams need to keep a laser-focus on the outcomes that are most important to decision-makers; the operational challenges of trials mean there is no room for ‘nice-to-haves’. The data that we have presented here suggest that ‘nice-to-haves’ are being collected, and work moved away from the focus of the trial: the primary outcome.

Trial burden was flagged in a 2019 multi-stakeholder James Lind Alliance Priority Setting Partnership on unanswered research questions in trial retention [19]. In this priority setting process, how to reduce burden on staff and participants was ranked the 3rd most important question, after how to make better use of existing data (2nd) and what motivates a participant to complete a trial (1st). The current study identified a total of 918 outcomes in just 161 trials, ranging from trials that collected a single outcome to one collecting 30 (Trial ID 152, a phase III drug trial). The burden that data collection often represents for staff and participants is likely to be a central factor in these questions getting their top 3 positions.

Staying with workload, it is worth remembering that ORINOCO focuses on data collection time, not total data collection effort. We do not know what spending three times as much time on secondary outcomes as on primary outcomes means for total data collection effort. We can, however, guarantee that total workload will be more than the time we quantify in this article. A good example of the distinction between data collection time and total data collection effort is the use of routine electronic medical record systems. If data exist in routine electronic systems, data collection time is 0 and data will instead be transferred from a data controller to the trial team for analysis. Achieving this transfer can be challenging. A recent UK study in two trials, Add-Aspirin and PATCH, found that it took 13 months for Add-Aspirin to receive data from the National Cancer Registration and Analysis Service and 15 months for PATCH to receive data from NHS Digital [20]. In another example, changing interpretation of information governance regulations by data controllers meant that the team behind the EPOCH trial were unable to gain access to post-discharge hospital data in Wales and had to change their primary analysis because of it [21]. Even in the extreme case of ‘*Time to collect*’ being 0.0, it is still important to consider total data collection effort. Total data collection effort will not be 0.0, but for the purposes of this project, we have ascribed the time as 0.0 minutes as we are focusing purely on data collection itself, rather than the time (and costs) associated with total data collection effort.

Core outcome sets—outcomes that should always be collected for a given type of trial—could help because they are developed using formal methods of patient and other stakeholder involvement to choose outcomes that do matter [9]. Our sample of trials using a core outcome set is small and the findings therefore far from definitive. However, they do at least suggest that for rheumatoid arthritis trials, using a core outcome set does not generally increase data collection workload compared to other phase III trials. Core outcome sets remain a minority choice, only 2% of all trials use them [22], although use in rheumatoid arthritis, the area we chose, is much higher with 82% of rheumatoid arthritis trials found to use a core outcome set in a recent systematic review [23]. Increased data collection workload does not appear to be an argument against using core outcome sets.

Core outcomes sets are unlikely to be enough though. It is important that discussions around outcome selection in trials include patients and other stakeholders, particularly those who will ultimately be tasked with collecting outcomes (e.g. research nurses). These stakeholders need to be the people making the healthcare decisions the trial is intended to inform: what outcome information do they need? Outcomes (and ways of measuring them) not on decision-makers’ lists are prime candidates for exclusion because they are judged unnecessary by those making the decisions. As mentioned previously, the ORINOCO project had three phases, and the third phase of work (not reported here) explores the process of who contributes to these discussions and how decisions about outcome selection and collection are made. There is a clear disconnect between outcome selectors (including patient and public contributors) and outcome collectors, which, at least in part, fuels the disconnect between what trialists want and what patients and health professionals want [16].

The picture for public health trials is different to that for the other trials. As Table 1 (minutes) and Table 2 (hours) show, public health trials focus proportionately more time on primary outcomes than phase III and core outcome trials but overall spend much more time on data collection. Public health trials generally have more participants and fewer secondary outcomes than the other trials in our sample, a combination that is likely to make data collection more expensive but more focused on primary outcomes.

### Strengths and limitations

There are a number of limitations. First, our sample of 161 trials is big but not *very* big. We were unable to get timing data for all trials. For phase III trials, this was perhaps not such a problem, but the number of core outcome set and public health trials was always modest, and then got smaller. Second, the core outcome set trials all looked at a single clinical area: rheumatoid arthritis. We are more confident about the general picture for phase III trials than we are for the two other trial types.

Third, it was difficult for many trial teams to say how long their data collection took. This is perhaps the greatest limitation because it means the timings we provide are sometimes uncertain. We think it is also something of a strength because it underlines our point that trial teams do not routinely give a great deal of thought to how long it will take to collect their trial outcomes. Even when teams have given it thought and were able to tell us how long each outcome took to collect, we did not find a single study that reported this in the trial publication. Given the well-known practical challenges of trials, it seems odd that there is no explicit attempt to assess the workload generated by the outcomes selected. Fourth, our calculation of total time assumed 100% retention at every measurement point, which is of course unlikely and means our times may be overestimates. This retention assumption does, however, reflect the maximum data collection workload a trial team has committed to in its design, which we think is worth knowing. Strengths are that we have not seen data similar to the data we present before, and all the included trials are recent: none were published earlier than 2015. The problem we highlight—that the majority of data collection effort is dedicated to the less important outcomes—is not a fading piece of history but very much relevant to the here and now.

### Implications for practice

This is simple: we think trial teams should routinely consider the work involved in collecting the data their selected outcomes need. They should then look at how this effort is distributed and decide whether for some outcomes that effort is not worth it given the relative importance of the outcome. That latter judgement about importance needs the explicit view of the intended users of the trial results, often patients and health professionals. Using a core outcome set will give greater confidence that selected outcomes are important. The trial budget should directly reflect the data collection workload. Our experience with ORINOCO has been that trial teams often have little or no idea how long their data collection took. This has to increase the chance of a mismatch between workload and available resources. Finally, trial teams could start to report (or at least monitor) how long data collection took, which would help them and others plan their own data collection. This has its own workload implications, and we should not forget that. But monitoring and reporting this information would allow trialists to design and budget appropriately for their data collection processes, from which we would anticipate efficiencies.

### Implications for research

Our database (https://osf.io/FNB3E/) is the starting point of a *Time to collect* tool that can help trial teams to estimate the data collection workload for their trial. Collecting more timing data would improve this tool, especially if done prospectively rather than retrospectively as was the case for our timing data. It would also be useful to know whether having a better idea of data collection workload during trial design does influence design decisions and budgeting. For example, knowing that a minor secondary outcome will demand substantial amounts of time (and therefore money) to collect ought to put a question mark next to inclusion of that outcome. Whether that is what happens would be interesting to know.

Core outcome sets suggest what is important to measure but not how to measure it. We think it would be useful for trialists to be more explicit about how to measure core outcomes. The configuration of some core outcome sets may be more efficient than others and it would be useful for trial teams (and funders) to know this when planning their trials. Finally, more work on the impact on total data collection effort of using routine data would be welcome. An additional challenge for trial teams will be that some ‘standard’ data collection items, such as health-related quality of life for cost-effectiveness evaluations, are unlikely to be available in routine data, which limits the scope for *Time to collect* reductions. Our data (see Supplementary files) do contain trials that used routine data, but we have not looked at these systematically.

## Conclusion

The majority of data collection time in trials is dedicated to the less important outcomes. Depending on trial type, between two and three times as much time is spent on secondary outcomes than on primary outcomes. Many trials teams have little idea how long their data collection takes, which will make it difficult to allocate resources effectively. Trial teams should explicitly consider how long it will take to collect the data for an outcome and decide whether that time is worth it given the relative importance of the outcome within the trial.

## Supplementary Information

**Additional file 1.**

**Additional file 2.**

**Additional file 3.**

**Additional file 4.**

**Additional file 5.**

## Data Availability

The bulk of our data are summarised in our Supplementary Files. All our data are available in https://osf.io/FNB3E/.

## Abbreviations

ID: Identifier
SF-36: 36-Item Short Form Health Survey
UK: United Kingdom
