# Supplementary material for: A good use of time? Providing evidence for how effort is invested in primary and secondary outcome data collection in trials
Source: Trials. 2022 Dec 27;23:1047. doi: 10.1186/s13063-022-06973-8 (PMC9793601; doi:10.1186/s13063-022-06973-8)
Supplement: Supplementary file 1 — Additional file 1. [file 13063_2022_6973_MOESM1_ESM.docx]

**Supplementary File 1**

Phase III trials

Run PubMed, 5 July 2019 (Note: an early search produced very few eligible studies. We modified the search after a discussion at a meeting of the project team).

("clinical trial"[Publication Type]) AND ("2014/01/01"[Date - Entrez] : "3000"[Date - Entrez])

‘Entrez’: date entered into PubMed

Total number of results: 152981

Core outcome set trials

Run May 2019

Trials meeting out eligibility criteria in the cohort of trials in Kirkham JJ, Clarke M, Williamson PR. A methodological approach for assessing the uptake of core outcome sets using ClinicalTrials.gov: findings from a review of randomised controlled trials of rheumatoid arthritis. BMJ 2017;357:j2262.

Total number of results: 21

Public Health trials

Run 18^th^ April 2019

(((preventive health services[MeSH Terms]) AND ((intervention*[Title/Abstract] OR program*[Title/Abstract] OR trial*[Title/Abstract]))) AND "randomized controlled trial"[Publication Type]) AND ("2014"[Date - Entrez] : "2019"[Date - Entrez])

Total number of results: 6803
